# Neonatal Early Onset Sepsis: Impact of Kaiser Calculator in an Italian Tertiary Perinatal Center

**DOI:** 10.1097/INF.0000000000003342

**Published:** 2021-09-09

**Authors:** Eleonora Pontello, Valentina Favero, Nicoletta Mainini, Francesca Tormena, Michela Giovannini, Beatrice Galeazzo, Anna Chiara Frigo, Paola Lago

**Affiliations:** From the * Neonatal Intensive Care Unit, Ca' Foncello Hospital, Treviso, University of Padua, Italy; † Department of Woman and Child Health, University of Padova, Padova, Italy; ‡ Department of Cardiac, Thoracic and Vascular Sciences, University of Padova, Padova, Italy.

**Keywords:** antibiotics, early onset sepsis, infection, newborns, sepsis calculator

## Abstract

Suspected early-onset sepsis (EOS) results in antibiotic treatment and blood withdraw of a substantial number of neonates who are uninfected. We evaluated if the EOS calculator can reduce antibiotic exposure and invasive procedures for suspected EOS in term and late preterm neonates, without any significant increase in adverse outcomes.

**Methods:**

The proportion of EOS risk in neonates ≥35 weeks gestation exposed to antibiotics, intensive monitoring and blood withdrawal was compared between a baseline period (January 2018–May 2018), when Centers for Disease Control guidelines approach was used, and a post-EOS calculator-implementation period (June 2018–December 2019).

**Results:**

We included 4363 newborn infants with gestational age ≥35 weeks, respectively 824 in baseline period and 3539 in the EOS calculator period. Among them, 1021 (23.4%) infants presented risk factors for neonatal sepsis. There was a halving in empirical antibiotics exposure: 3% in the baseline and 1.4% in the post-EOS-implementation period, *P* < 0.05. Blood culture and laboratory evaluations had fallen from 30.6% to 15.4% (*P* < 0.05). Close monitoring of vital parameters decreased from 25.4% to 4.8% (*P* < 0.05). The number of antibiotic days per 100 live births decreased from 15.05 to 6.36 days (*P* <0.05). The incidence of culture-confirmed sepsis and clinical sepsis was very low in 2 periods. Only one infant identified at low-risk by Kaiser calculator at birth developed symptoms after 12 h from birth. We had no readmissions for EOS.

**Conclusions:**

Application of the EOS calculator more than halved the burden of intensive monitoring and antibiotic exposure, without compromising safety in a population with a relatively low incidence of culture-proven EOS and good access to follow-up care.

Neonatal early-onset sepsis (EOS) is a serious and potentially fatal complication of birth. It is defined as an invasive bacterial infection of the blood and/or cerebrospinal fluid (CSF) that occurs in the first 72 h of life. The pathogenesis is primarily ascending colonization of the maternal genital tract and uterine compartment by normal maternal gastrointestinal and genitourinary tract bacterial flora, resulting in subsequent colonization and infection of the fetus or newborn. Group B Streptococcus (GBS) and *Escherichia Coli* are the most common bacteria involved.^1^ Maternal risk factors for neonatal sepsis in both term and late-preterm infants include chorioamnionitis, intrapartum maternal temperature ≥38°C, delivery at <37 weeks’ gestation, maternal GBS colonization and prolonged rupture of membranes (ROM) (≥18 h).^2^

Neonatal EOS declined substantially over the last two decades, primarily due to the implementation of evidence-based maternal GBS screening and intrapartum antibiotic prophylaxis.^3^ Although the incidence of sepsis in this population is low (approximately from 0.5 per 1000 live births at term to 1 per 1000 live births among late-preterm infants), potentially serious adverse outcomes, including death, ensure that caregivers use a low threshold for evaluation and treatment of possible sepsis.^4^ However, there is also recognition that we must prevent antibiotic overuse to avoid risks and costs for newborns. Needless antibiotic therapy leads to maternal-infant separation, parental anxiety, increased health expenditures, emergence of antibiotic resistance and adverse outcomes.

The Centers for Disease Control (CDC) and Prevention 2010 guidelines to preclude neonatal GBS disease provided algorithms to manage neonatal EOS, with laboratory evaluation (complete blood count, [CBC] and blood culture) and empiric antibiotic treatment for 48 h in infants born to mothers with chorioamnionitis.^3^

Recently, continued adherence to CDC guidelines using a categorical risk factor assessment, despite declining incidence of EOS, raised concerns about over-investigation and over-treatment with antibiotics, with a series of negative consequences including unnecessary neonatal intensive care unit (NICU) admission, separation of mother and baby, difficult breast-feeding, increased hospital costs and antibiotic resistance.

The management of term and late-preterm newborns at risk of EOS, when they were not clearly symptomatic, during the last few decades remains controversial.^5^

The CDC reports that 25%–50% of all antibiotics prescribed in the US are unnecessary, with alarming consequences of antibiotic resistance and risk of toxicity.^6^ Thus, evidence-based strategies to reduce antibiotic exposure are badly needed.

A reappraisal of guidelines to manage infants with EOS, advocates close observation of healthy-appearing infants ≥35 weeks gestational age, born to mothers diagnosed with EOS-risk factors, rather than empiric antibiotic treatment.^2^

Researchers at Kaiser Permanente Northern California developed a multivariate predictive model to estimate the risk of EOS in infants 35 weeks and older in terms of gestational age. The neonatal EOS calculator is a tool for instructed clinicians to guide standardized management of EOS, currently endorsed as a possible strategy by the American Academy of Pediatrics, a viable alternative to categorical risk evaluation approach or serial physical examinations of infants (SPEs).^2^

We evaluated efficacy, safety and clinical applicability of the neonatal EOS risk calculator for suspected EOS in our third level perinatal center as part of a quality improvement initiative.

## METHODS

### Study Design, Setting and Population

This is a retrospective observational study, comparing 2 periods before and after the EOS calculator introduction in all infants, born at 35 weeks or later at Treviso Hospital. We excluded 34 weeks’ gestation infants because at our institution, they are routinely admitted to the NICU and experience a higher level of monitoring. The baseline period was defined from January 1, 2018, through May 31, 2018, prior to introducing the EOS calculator, when clinical management was based on CDC GBS guidelines. The indications contained in our precalculator policy are listed in Table 1.

**TABLE 1.** Pre-calculator policy during baseline period

Clinical examination and/or EOS risk factors	Description of local pre-calculator policy
Symptomatic newborn	Blood culture, laboratory evaluations (CBC count, PCR) and empirical antibiotics.
Newborn born from mother with suspected chorionamnionitis as by obstetrician, even if asymptomatic	Blood culture, laboratory evaluations (CBC count, PCR) and empirical antibiotics.
Well appearing newborn with positive or unknown maternal GBS status and none or incomplete (<4 h prior to delivery) intrapartum antibiotic prophylaxis with one of the following additional risk factors:	Blood culture, laboratory evaluations (CBC count, PCR), close monitoring of vital signs (heart rate, respiratory rate, SatO_2_, body temperature and skin color) every 8 h for 48 h by nurses and clinical monitoring for 48 h.
Duration of rupture of membranes >18 h	
Gestational age <37 weeks.	
Well appearing newborn with positive or unknown maternal GBS status and none or incomplete (<4 h prior to delivery) intrapartum antibiotic prophylaxis with one of the following:	Close monitoring of vital signs (heart rate, respiratory rate, SatO_2_, body temperature and skin color) every 8 h for 48 h by nurses and clinical monitoring for 48 h.
Duration of rupture of membranes <18 h	
Gestational age >37 weeks	
Well appearing newborn with positive maternal GBS status with complete (>4 h prior to delivery) intrapartum antibiotic prophylaxis	Routine monitoring for 48 h. Discharge after at least 48 h.
Asymptomatic newborn without EOS risk factors	Routine care

CBC, complete blood count; EOS: early onset sepsis; GBS, group B streptococcus.

The application period spanned June 1, 2018, to December 31, 2019, when the calculator was an integral part of the electronic medical record. During the post-calculator period, we shortened the timing of close monitoring of vital signs (heart rate, respiratory rate, SatO_2_, body temperature and skin color) by nurses from 8 to 4 h. A complete neonatal clinical examination is scheduled every 12 h.

The primary goal was to evaluate the impact of the EOS calculator on antibiotic use in the first 72 h of life, in infants with suspected EOS. The secondary outcome was to assess the need for close monitoring of vital signs (heart rate, respiratory rate, body temperature and skin color every 4 h) and a blood draw (blood culture and CBC count). We evaluated differences in the number of antibiotic days per 100 live births.

The EOS calculator is an accurate multivariate predictive model of risk to establish prior probability for newborn sepsis, based on objective data at birth, which could be combined with a neonatal physical examination to rate posterior probability for clinical management (observation, blood tests and empirical treating). Key risk factors that determine prior EOS probability are gestational age, highest maternal antepartum temperature, GBS carriage status, duration of ROM and type and timing of intrapartum antibiotics.^7^

Neonatal EOS calculator and classification of the infant’s clinical presentation are available online at https://neonatalsepsiscalculator.kaiserpermanente.org.

We instructed clinicians and nurses with interactive meetings to screen all newborns with the EOS calculator to become confident with this new tool. Instructed nurses calculated online the estimated risk of EOS at birth and reported the result of the assessment in the local electronic medical record. The web platform is structured for identifying and ranking all factors critical to the decision, so the nurses themselves were able to recognize EOS risk factors and different clinical presentations (well-, equivocal- and clinically ill-appearing newborn). In case of a well-appearing newborn with maternal EOS risk factors, the nurse has been instructed to use the calculator at birth and alert the neonatologist in case of need to monitor vital parameters or taking blood tests or an increased risk for EOS. The neonatologist verified the EOS risk evaluation and clinical management chosen at birth or during the first clinical examination. In addition, she/he verified EOS calculator accuracy and compliance.

The study was approved by the local Ethics Committee.

### Data Collection

Demographic data, procedures and discharge diagnosis codes were collected for the entire birth cohort. Maternal data included delivery mode, GBS status, duration of ROM, maternal intrapartum temperatures and intrapartum antibiotics prophylaxis.

Data from neonatal medical records included sex, gestational age, birth weight, Apgar score, a need for close monitoring of vital signs and sepsis screening, results of blood and CSF cultures, CBC count and the C-reactive Protein (CRP), plus antibiotic treatment.

By convention, the CDC EOS incidence of 0.5/1000 live births were used to compute the sepsis risk calculator. We defined EOS as blood or CSF culture-confirmed infection with a pathogenic bacterial species that occurred from birth through the first 72 h of life. We defined clinical sepsis with the following CDC criterion: patient ≤1 year of age has at least 1 of the following clinical signs or symptoms with no other recognized cause: fever (>38°C rectal), hypothermia (<37°C rectal), apnea or bradycardia AND blood culture not done or no organisms detected in blood AND no apparent infection at another site AND physician institutes treatment for sepsis.^8^

### Statistical Analysis

Dichotomous variables were summarized with the number and percentage of infants in each category, continuous variables with mean and standard deviation (SD).

Comparison of infant and maternal characteristics during the two periods were analyzed with χ^2^ or Fisher’s exact test in case of dichotomous variables, with Student’s *t* test for continuous ones. Outcomes were compared considering a binomial distribution for antibiotic therapy, blood testing, close monitoring of vital parameters and monitoring in the NICU, and a Poisson distribution for duration of antibiotic therapy with the Wald χ^2^ test and the odds-ratio with 95% confidence interval (CI).

The statistical significance was established to be *P* < 0.05, while data were analyzed with SAS 9.4 (SAS Institute Inc., Cary, NC, USA) for Windows.

## RESULTS

The study cohort included 4363 newborn infants with gestational age ≥35 weeks, respectively, and 824 in the baseline period, including 3539 in the EOS calculator period. Among them, 1021 (23.4%) infants presented maternal risk factors and/or clinical symptoms for neonatal sepsis, including 211 (25.6%) in the baseline period and 810 (22.9%) in the EOS calculator period. Infant characteristics born in these 2 periods were similar for sex, gestational age, birth weight, Apgar score, delivery method and maternal risk factors. (Table 2)

**TABLE 2.** Infant characteristics by study period

Infant Characteristics	Total life births 01/01/18 to 31/12/19 (N = 4363)	Pre-EOS 01/01/18 to 31/05/18 (N = 824)	Post-EOS 01/06/18 to 31/12/19 (N = 3539)	*P* value
BW gr mean (SD)	3313.2 (490.7)	3300.4 (503.3)	3316.2 (487.6)	0.40*
GA weeks mean (SD)	39.1 (1.5)	39.1 (1.5)	39.1 (1.4)	0.89*
Male N (%)	2261 (51.8)	440 (53.4)	1821 (51.5)	0.32†
Apgar index 1’ mean (SD)	8.8 (1)	8.8 (0.9)	8.7 (1)	0.77*
Apgar index 5’ mean (SD)	9.8 (0.6)	9.8 (0.6)	9.8 (0.6)	0.75*
Cesarian section N (%)	996 (22.8)	202 (24.5)	794 (22.4)	0.20†
EOS-risk e/o symptomatic newborn ≥35 weeks N (%)	1021 (23.4)	211 (25.6)	810 (22.9)	0.10†
Maternal EOS-risk factors N (%)	961 (22.0)	201 (24.4)	760 (21.5)	0.07†
Symptomatic newborn ≥ 35 weeks N (%)	149 (3.4)	28 (3.4)	121 (3.4)	0.98†
Intrapartum maternal temperature ≥38°C N (%)	47 (1.1)	13 (1.6)	34 (1.0)	0.12†
Maternal GBS colonization N (%)	643 (14.7)	126 (15.3)	517 (14.6)	0.62†
PROM (≥18 h) N (%)	273 (6.25)	16 (1.9)	257 (7.3)	<0.001†

* Student’s *t* test.

† χ^2^ test.

BW indicates birth weight; EOS: early onset sepsis; GA, gestational age; GBS, group B streptococcus; N, number; PROM, prolonged rupture of membranes.

After implementing the new management strategy, there was a 54% relative reduction in the proportion of neonates exposed to antibiotics in the first 72 h, declining respectively from 3% to 1.4% (*P* < 0.05). Blood culture and laboratory evaluations fell from 30.6% to 15.4% (*P* < 0.05). Close monitoring of vital parameters decreased from 25.4% to 4.8% (*P* < 0.05). Monitoring in the NICU decreased from 6.2% to 4.5% (*P* < 0.05). The number of antibiotic days per 100 live births decreased from 15.05 to 6.36 days (*P* < 0.05). The incidence of culture-confirmed EOS was very low across the 2 periods. (Table 3)

**TABLE 3.** Clinical outcomes by study period

Clinical Outcomes	Total N (%) (N = 4363)	Pre-EOS N (%) (N= 824)	Post-EOS N (%) (N = 3539)	*P* value	OR (95% CI)
EOS-risk e/o symptomatic newborn ≥35 weeks N (%)	1021 (100)	211 (25.6)	810 (22.9)	0.10*	0.86 (0.72–1.03)
Close monitoring of vital parameters N (%)	379 (8.6)	209 (25.4)	170 (4.8)	<0.001*	0.15 (0.12–0.19)
Monitoring in NICU N (%)	210 (4.8)	51 (6.2)	159 (4.5)	0.04*	0.71 (0.52–0.99)
Blood tests N (%)	193 (4.4)	66 (30.6)	127 (15.4)	<0.001*	0.42 (0.30–0.57)
Culture confirmed sepsis N (%)	3 (0.068)	2 (0.242)	1 (0.028)	0.08*	0.12 (0.01–1.28)
Clinical suspected sepsis N (%)	7 (0.160)	3 (0.364)	4 (0.113)	0.13*	0.31 (0.07–1.39)
Low-risk infants who develop EOS N (%)	–	–	1 (0.028)	–	–
Antibiotic use in the first 72 h N (%)	74 (1.6)	25 (3.0)	49 (1.4)	0.001*	0.45 (0.28–0.73)
Antibiotic use days (number of antibiotic days per 100 live births)	349 (8.0)	124 (15.1)	225 (6.4)	<0.001†	0.42 (0.34–0.53)

* Wald χ^2^ test using binomial distribution.

† Wald χ^2^ test using Poisson test.

CI indicates confidence interval; EOS, early-onset sepsis; NICU, neonatal intensive care unit.

**FIGURE 1.**
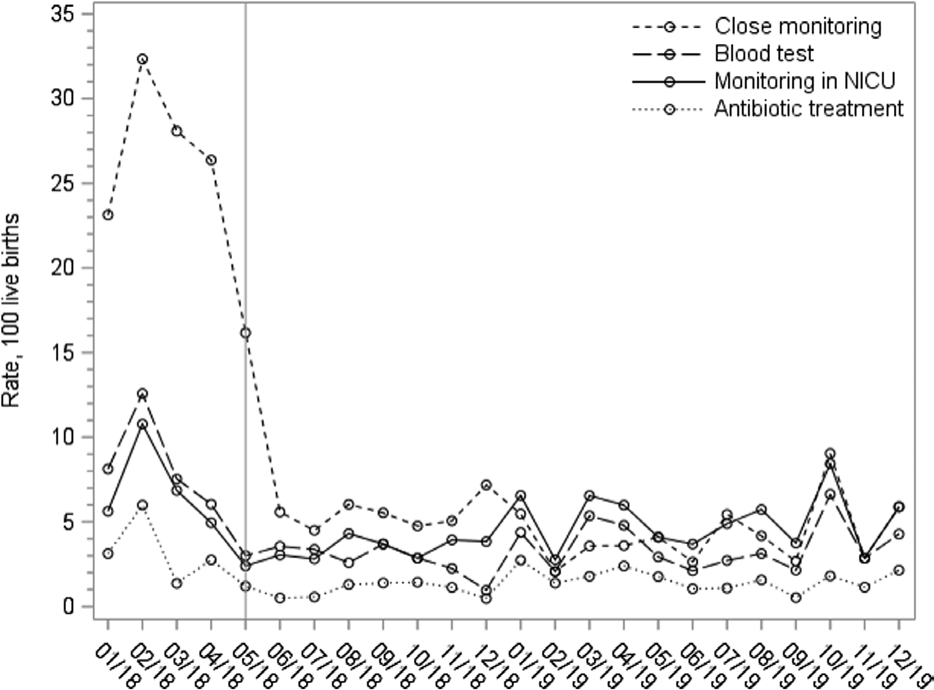
Clinical Outcomes by Study Period. This figure shows the trajectories of these reductions starting immediately after the introduction of the new management strategy and continued in the EOS calculator period.

Figure 1 shows the trajectories of these reductions, starting immediately after the introduction of the new management strategy and continuing in the EOS calculator period.

We had no readmissions for EOS or cases of infection-attributable deaths.

## DISCUSSION

This quality improvement initiative aimed to reduce unnecessary antibiotic exposure in term and late-preterm infants at risk for EOS.

“Rule-out sepsis” remains one of the common clinical tasks conducted by neonatologists. The challenges for clinicians are 3-fold: promptly identifying neonates with a high likelihood of sepsis and initiating antimicrobial therapy; distinguishing between “high-risk” healthy-appearing infants and infants with clinical signs who did not require treatment; this also included discontinuing antimicrobial therapy once sepsis was deemed unlikely.

Neonatologists are concerned about initially well-appearing infants with identified risk factors for EOS, in fear of missing the opportunity to intervene before infants become critically ill. Ill-appearing infants are treated with empirical antibiotics, after obtaining blood tests and cultures. For equivocal and well-appearing at-risk infants, management can vary. Options include close clinical monitoring, screening laboratory assessments (CBC and CRP or procalcitonin), blood culture and empirical antibiotics.^9^ This arbitrary decision is made by the attending physician, based on the combination of maternal EOS risk factors, physical examination and/or results of the CBC and CRP. This approach may expose newborns to unnecessary laboratory sepsis screens and antibiotic therapy.

A more rational approach may be the use of a calculator to assess the risk of EOS for the evaluation of a healthy-appearing newborn. It was developed by researchers at Kaiser Permanente Northern California as a multivariate predictive model to estimate the risk of EOS in infants 35 weeks and older’ gestation. It incorporated two linked predictive models, using the Bayesian approach. The first model considered the prior probability of EOS, based on gestational age, maternal GBS status, duration of ROM, highest antepartum maternal temperature and timing and type of intrapartum antibiotics. The second model evaluated how the baseline risk was modified by neonatal examination. Investigators reported that the sepsis calculator was associated with a significant reduction in antibiotics for neonates, without adverse outcomes, for example mortality from infection or readmission.^10^

The present retrospective study compared the more traditional approach, based on CDC EOS recommendations, using a multivariate risk assessment.

In our study we found that 23.4% of newborns presented maternal EOS risk factors and/or symptoms; other studies reported an incidence of newborns with EOS risk factors, respectively, of 11%^11^ and 16.8%,^12^ with certain differences in ranking risk factors. According to what was detected in the baseline period, the strategy focused on categorical risk factors which were likely to result in unnecessary medical interventions in uninfected infants. Instead, we demonstrated that a multivariate approach, based on the EOS calculator, was effective without compromising safety. In fact, the use of the EOS-calculator has allowed to better stratify the risk in well-appearing newborns born from mothers with clinical signs of suspected chorionamnionitis and to reduce unnecessary antibiotic therapy in newborns with “altered” blood tests and nonspecific clinical manifestations, potentially associated with the normal transition to extrauterine life.

In the present study, its application was associated with a reduction in antibiotic therapy by more than 50%, with relevant consequences for the emergence of antibiotic resistance and adverse effects. We also found a statistical and clinically significant reduction in blood tests from 30.6% to 15.4% and close monitoring of vital parameters from 25.4% to 4.8%, and NICU admissions from 6.2% to 4.5%. These aspects also coincided with a reduction in nursing overload. The large drop in close clinical monitoring could be explained by two key components of the EOS calculator: a better risk stratification and a targeted monitoring in EOS-risk infants.

In line with our data, a meta-analysis revealed a relative risk of antibiotic use of 56% (95% CI: 53–59%) in before and after studies that included newborns, regardless of exposure to chorioamnionitis.^13^

Our findings are similar to those reported in previous studies, which included newborns 35 weeks and older’ gestation; nevertheless, we showed a lower rate of antibiotic use.^10,11,13–15^ Kuzniewicz et al^10^ found a decrease of blood cultures in the first 24 h, from 14.5% to 4.9%, and a reduction of empirical antibiotic administration in the first 72 h, from 5.5% to 3%. Achten et al^11^ reported that empirical treatment of EOS was reduced from 4.8% to 2.7% after sepsis calculator implementation. Strunk et al^14^ showed a decrease of blood culture from 15.2% to 11.1%, and a reduction of empirical antibiotic administration from 13.7% to 8.2% in the week after birth. Dhudasia et al^15^ found a decrease of blood culture from 26.9% to 4.9% and a reduction in antibiotic therapy of 42% (from 6.3% to 3.7%) in newborns <72 h old.

For only term infants (>37 weeks) at risk for EOS, Eason et al^12^ showed a decrease from 63% to 3%, with no evidence of infection in those receiving a 36-h course of antibiotics, and from 31% to 5% in infants with suspected infection receiving a 5-day course.

Some studies reported efficacy of the approach among preterm newborns at 34 weeks’ gestation, not included in our evaluation due to local protocol. Arora et al^6^ reported a significant reduction in sepsis evaluations (from 90.9% to 68.8%) and a reduction in antibiotic therapy by 29.4% in infants ≥34 weeks’ gestation admitted to the NICU. Akangire et al^16^ showed a decrease of blood cultures from 14.8% to 7.6%, and a reduction of empirical antibiotic administration from 11% to 5% in neonates at 34 weeks’ gestation or more.

Implementation of the EOS calculator decreased unnecessary painful procedures, while NICU admissions enabled healthy-appearing infants to remain in the newborn nursery with their mothers, and as such promoted bonding.^6,10–18^ In these studies, the EOS incidence was similar between study periods, and no safety concerns were identified.

Our project was implemented without any extra cost or staffing and was able to facilitate a cost reduction due to less NICU admissions, procedures and antibiotic use. The tool appeared easy-to-use and was well-accepted by nursing staff, who appreciated the time savings compared with the previous approach.

This is not the only useful strategy. According to the new American Academy of Pediatrics guidelines, some studies sustained the EOS risk assessment by performing SPEs over the first 24 to 48 h of life.^17,19–21^ This strategy is based on close observation of neonates with mild or equivocal symptoms during their first few hours (ie, neonates born by cesarean section with mild tachypnea that resolves spontaneously within a few hours). Each examiner fills in and signs a standardized form (detailing general well-being, skin color, including perfusion and the presence of respiratory signs) at standard intervals (at 3-6-12-18-36-48 h). Nursing staff and midwives notify clinicians when signs of illness develop. The SPEs strategy reduces unnecessary laboratory evaluations and antibiotics, and does not worsen outcomes of neonates at-risk or neonates with mild, equivocal or transient symptoms. Vatne et al^17^ found a 57% relative reduction in term newborns exposed to antibiotics (from 2.9% to 1.3%) and a 60% relative reduction in mean antibiotic therapy-days/1000 patient-days, by SPEs. This approach cannot be adapted to our setting, as it requires necessary human resources.

## LIMITATIONS

We reported only one center’s experience, coupled with a relatively short pre-intervention period. Due to a lack of local data for EOS incidence in our neonatal population, we used the CDC incidence of EOS (0.5/1000 live births) as a pretest probability for the EOS calculator. As a retrospective study, in the baseline period, some maternal data related to GBS status and intrapartum antibiotics prophylaxis were incomplete in the electronic record, so could not be accurately collected. After the introduction of the EOS calculator, maternal data were systematically indicated in the medical record, part of EOS-risk parameters.

We could not exclude that some neonates discharged home have been readmitted elsewhere. However, Treviso Hospital is a reference hospital for the entire province that receives the vast majority of neonates.

### Conclusion

Application of the EOS calculator halved the burden of intensive monitoring and antibiotic exposure, without compromising safety in a population with a relatively low incidence of culture-proven EOS, and access to follow-up care. We reported a significant reduction in sepsis evaluations and antibiotic therapy, with no safety concerns identified. It is encouraging to reduce unnecessary sepsis evaluations and antibiotic therapy, with a positive effect on antibiotic resistance, drug-related adverse events, and health expenditures, which included the preservation of maternal-infant bonding.

## ACKNOWLEDGMENTS

The authors acknowledge all physicians and nurses of the Perinatal Centre and Neonatal Intensive Care Unit of Treviso, Italy, for support, advice, and commitment to providing high-quality care for these young patients and their families.

The study was approved by local ethic committee as well as the treatment of patients’ data.

All clinical data are available and stored on our server at the Neonatal Intensive Care Unit, Azienda ULSS 2 Marca Trevigiana, Treviso, Italy.
